# ALDH1 might influence the metastatic capability of HeLa cells

**DOI:** 10.1007/s13277-015-3398-y

**Published:** 2015-04-13

**Authors:** Tingting Yao, Rongbiao Lu, Yiqing Li, Yongpai Peng, Miao Ding, Xiaofei Xie, Zhongqiu Lin

**Affiliations:** 1 Department of Gynecological Oncology, Sun Yat-sen Memorial Hospital, Sun Yat-sen University, 107 Yan Jiang West Road, Guangzhou, 510120 China; 2 Key Laboratory of Malignant Tumor Gene Regulation and Target Therapy of Guangdong Higher Education Institute, Sun Yat-sen University, 107 Yan Jiang West Road, Guangzhou, 510120 China; 3 Department of Dermatology, Third Affiliated Hospital, Sun Yat-sen University, Guangzhou, Guangdong China; 4 Department of Hematology, Sun Yat-Sen Memorial Hospital of Sun Yat-Sen University, Guangzhou, China

**Keywords:** ALDH1, siRNA, Recombinant plasmid, Cervical cancer, HeLa

## Abstract

Recent data suggest that tumor persistence and recurrence could be caused by the presence of cancer stem cells (CSCs). Aldehyde dehydrogenase 1 (ALDH1) has been implicated in cancer pathogenesis and used as a CSC marker. We previously reported that cervical carcinoma contains a small subpopulation of cells expressing ALDH1 [1]. In this study, we used small interfering RNA to suppress ALDH1 expression and introduced an ALDH1 reporting vector into HeLa cells followed by various in vitro assays. We showed that knockdown of ALDH1 expression reduced the cell migration ability of HeLa cells, whereas augmented expression of ALDH1 increased cell migration. However, there was no difference in the cellular proliferation, apoptosis, cell cycle, and invasion. These results indicate that ALDH1 is directly involved in HeLa migration.

## Introduction

Cervical cancer is the third most common cancer in women worldwide and the most frequently occurring cancer of the female reproductive tract [2]. HPV infection has been shown to play a critical role in its etiology; however, it is not sufficient. Despite recent advances in the early detection and diagnosis of cervical cancer, the incidence is increasing and current treatments are unsatisfactory.

A number of studies have shown that a small proportion of tumor cells, designated as cancer stem cells, were capable of self-renewal and played a decisive role in tumor formation and growth [3, 4]. Additionally, tumor recurrence after conventional therapy has been suggested to result from the presence of these slow-cycling cells, which are capable of initiating and sustaining neoplastic growth [5, 6]. The resistance of cancer stem cells (CSCs) to conventional chemotherapy and radiotherapy has been attributed to cellular mechanisms such as multidrug resistance, quiescence, enhanced DNA repair abilities, and anti-apoptotic mechanisms [7]. Dysregulation of key signaling pathways such as the Wnt, Hedgehog, Notch, and TGF-β/BMP pathways plays an important role in modulating tumorigenesis as well as in stem cell function [8]. These studies suggest that identification and targeting cancer stem cells are key aspects to optimizing cancer therapy. CSCs have been isolated and cultured as structured spheroids in the presence of serum and growth factors. Several markers have been proposed to identify and isolate cancer stem cells including CD133, CD44, CD24, CD90, CD34, CD117, CD20, and aldehyde dehydrogenase (ALDH1) [7, 9], a cytosolic enzyme that is responsible for the oxidation of retinaldehydes to retinoids [10, 11].

Some members of the ALDH super-family play key roles in the enzymatic detoxification of endogenous and exogenous aldehydes and in the formation of important metabolic molecules such as retinoic acid or gamma-aminobutyric acid [12]. Thus, ALDH gene mutations lead to defective metabolism and provide the basic features of diseases such as gamma-hydroxybutyric aciduria, pyridoxine-dependent seizures, Sjögren syndrome, or type II hyperprolinemia [13]. Recent studies have shown that ALDH1 is a cancer stem cell marker and that its presence strongly correlates with tumor malignancy and self-renewal properties of stem cells in different tumors, including breast cancer, hepatoma, and colon and lung cancer [14–17]. We have previously reported that expression of ALDH1 was found in 24.77 % of cervical carcinoma by immunohistochemical staining. Additionally, flow cytometric analysis, qRT-PCR, and Western blot confirmed the presence of small subpopulations of ALDH1-positive cells. To examine the significance, we regulated and monitored ALDH1 gene expression by real time (RT)-PCR and Western blotting in HeLa cells. There was no change in the proliferation, apoptosis, and cell cycle after alteration of ALDH1 expression. Additionally, we demonstrated that reduction of ALDH1 led to markedly decreased migration, whereas restoration improved migration, suggesting that ALDH1 might potentially function as a marker of cervical CSC.

## Materials and methods

### Cell culture

The HeLa human cervical adenocarcinoma cell line was purchased from the American Type Culture Collection and maintained in RPMI 1640 medium (Life Technologies) supplemented with 10 % fetal bovine serum (Invitrogen).

### siRNA transfection

Three small interfering RNAs (siRNAs) targeting specific sequences of ALDH1 (NM_017617) and a scrambled siRNA (not homologous to any gene) as a negative control were synthesized by GenePharma (Shanghai, China). Searches of the genome database (BLAST, SSEARCH) were conducted to ensure that these sequences would not target other genes. Preliminary experiments indicated that, of the three siRNAs, the siRNA targeting the specific sequence (sense′-CCAAAGUCCUGGAGGUUGAdTdT-3′ and antisense 3′-dTdTGGUUUCAGGACCUCCAACU-5′) yielded the greatest downregulation of ALDH1 expression (data not shown). This siRNA was selected for further investigation. A non-targeting siRNA (nt-siRNA), which did not target any known gene, was used as a negative control. We performed siRNA transfection efficiency assays using varying concentrations of siRNA and determined that 25 nmol of siRNA provided optimal transfection efficiency (data not shown). In each experiment, the nt-siRNA transfection, particle stimulation-only groups (nontransfection, particle stimulation only), and the negative control (NC = nontransfection and nonparticle stimulation) groups were included as controls. HeLa cells were seeded into 6-well plates (4–5 × 10^4^ cells/well) and cultured in 2 mL of basic culture medium containing 10 % FBS until the cells were 70 % confluent. The siRNA-Lipofectamine™ 2000 complex was pre-mixed according to the manufacturer’s instructions and added to the 6-well plates. The results were confirmed by real-time RT-PCR.

### Plasmids and primers

Human ALDH1 cDNA was cloned into the pIRES2-vector (Clontech, Santa Clara, CA) between the BamHI and the HindIII restriction sites (pIRES2/ALDH1), and a corrective construct was verified by DNA sequencing. The pIRES2/ALDH1 construct was transfected into HeLa cells using Lipofectamine plus reagent (Invitrogen, San Diego, CA) following the manufacturer’s instructions. The cells were cultured in the presence of 1 mg/mL of G418 to establish stable cell lines. The expression of ALDH1 was confirmed by Western blot and reverse transcriptase polymerase chain reaction.

### Quantitative real-time PCR

All the reactions were performed in a 20-μL reaction volume in triplicate by SYBR Green Real-time PCR Universal Reagent (GenePharma Co., Ltd.) and MX-3000P Real-time PCR equipment (Stratagen). Standard curves were generated, and the relative amount of ALDH1 was normalized to 18S RNA (2^−ΔΔCt^). The ALDH1 expression fold change was evaluated using 2^−ΔΔCt^. The primers are ALDH1 (forward, 5′-CTGCTGGCGACAATGGAGT-3′; reverse, 5′-GTCAGCCCAACCTGCACAG-3′) and 18S rRNA (forward 5′-CCTGGATACCGCAGCTAGGA-3′ and reverse 5′-GCGGCGCAATACGAATGCCCC -3′). These primers yielded 230- and 112-bp products, respectively.

### Cell proliferation assays

The cell survival was assessed using the 3-(4,5-dimethylthiazol-2-yl)-5-(3-carboxymethoxyphenyl)-2(4-sulfophenyl)-2H-tetrazolium (MTS) assay (Sigma). For the MTS assay (Promega), 1 × 10^4^ cells/well were seeded in triplicate in a 96-well plate for each cell line. At 12, 24, and 48 h, 20 μL of MTS reagent was added to each well and incubated for 1 h at 37 °C; the results were analyzed by a plate reader at 490 nm. The sample data were normalized to the background readings of media only.

### Cell cycle analysis

Forty-eight hours after transfection, the cells were harvested and fixed in 70 % ice cold ethanol and followed by RNase A treatment, stained with 50 lg/mL of propidium iodide for the DNA content analysis by flow cytometry on a FACS Calibur system (EPICS ALTRA, Beckman Coulter, Fullerton, CA). The data were collected and processed using FlowJo FACS analysis software (Tree Star, Ashland, OR).

### Apoptosis assays

The cell pellets were resuspended in Annexin V-FLUOS staining solution (Roche Molecular Biochemicals) and incubated for 15 min at room temperature. The samples were then analyzed on a FACS Calibur system (EPICS ALTRA, Beckman Coulter, Fullerton, CA).

### Transwell cell migration assay

Forty-eight hours after transfection, the cells were resuspended in serum-free RPMI 1640 medium, and 5 × 10^4^ cells were added to the upper chamber of Matrigel-coated 24-well plates (8-μm pore size, Corning, Corning, NY). The lower compartment was filled with RPMI 1640 medium containing 20 % FBS. After 24 h, the cells that had not migrated were removed from the upper face of the filters using cotton swabs, and the cells that had migrated to the lower surface of the filters.

### Statistical analyses

The normally distributed continuous variables were compared by a one-way analysis of variance (ANOVA). When significance between the groups was apparent, multiple comparisons of the means were performed using the Bonferroni procedure with type I error adjustment. The data are presented as the means ± standard deviation (SD). *P* values of less than 0.05 were deemed significant. Repeated measurements with a linear mixed model were conducted to determine the group effect in the change of the MTS OD values among the groups. The statistical assessments were two-sided and evaluated at the 0.05 level of significant difference. The statistical analyses were performed using SPSS 15.0 statistical software (SPSS, Inc., Chicago, IL).

## Results

### ALDH1 gene expression in HeLa cells was successfully disturbed by transfecting siRNA or overexpressing plasmid

**Fig. 1:**
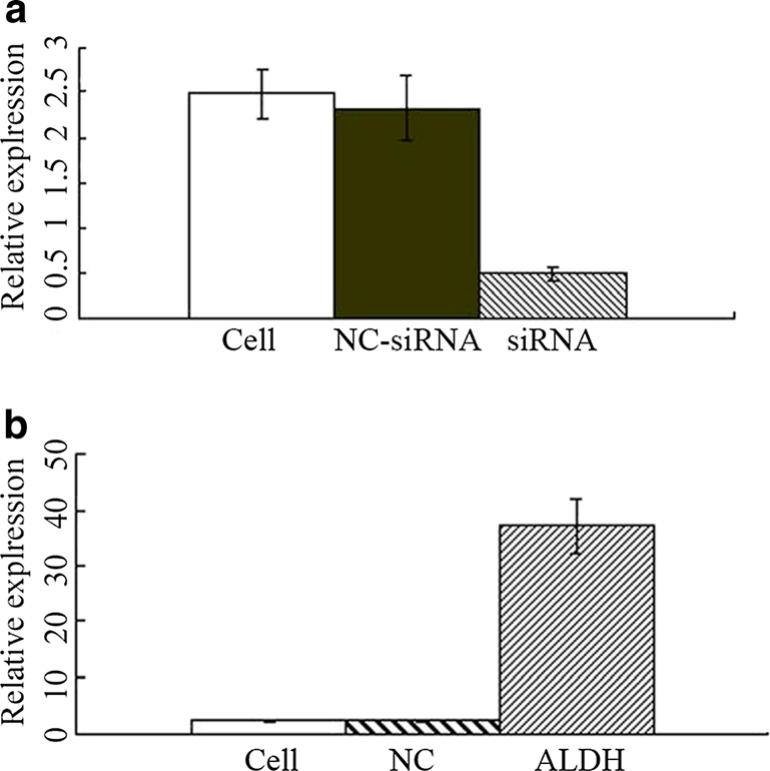
Expression of *ALDH1* in various cells. **a** The mRNA expression of *ALDH1* in the cells without treatment, the cells with non-targeting siRNA and the cells with siRNA targeting the specific sequence. **b** The detection of *ALDH1* mRNA levels in cells without any treatment, the cells just with vector and cells with a pIRES2/ALDH1 construct

RNAi technology was employed to silence the ALDH1 expression. Real-time PCR and Western blot were performed to quantify ALDH1 expression. Endogenous ALDH1 mRNA expression (Fig. 1a) were significantly lowered by RNAi (HeLa-ALDH1-RNAi) than by the control transfections (HeLa-NCi) and HeLa cells. To increase the ALDH1 expression, the cells were stably transfected with an ALDH1 expression construct. After transfection and G418 selection, the ALDH1 mRNA (Fig. 1b) expression levels in the stably transfected cells (HeLa-ALDH1) were much higher than in the HeLa cells.

### No effect of ALDH1 on proliferation, apoptosis, and cell cycle

**Fig. 2:**
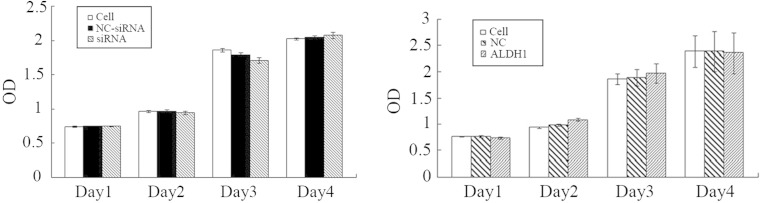
The MTT assay to evaluate cell proliferation. **a** The OD value of the cells without any treatment, the cells with non-targeting of siRNA and the cells with siRNA targeting the specific sequence. **b** The OD value of the cells without treatment, the cells with vector and the cells with a pIRES2/ALDH1 construct

**Fig. 3:**
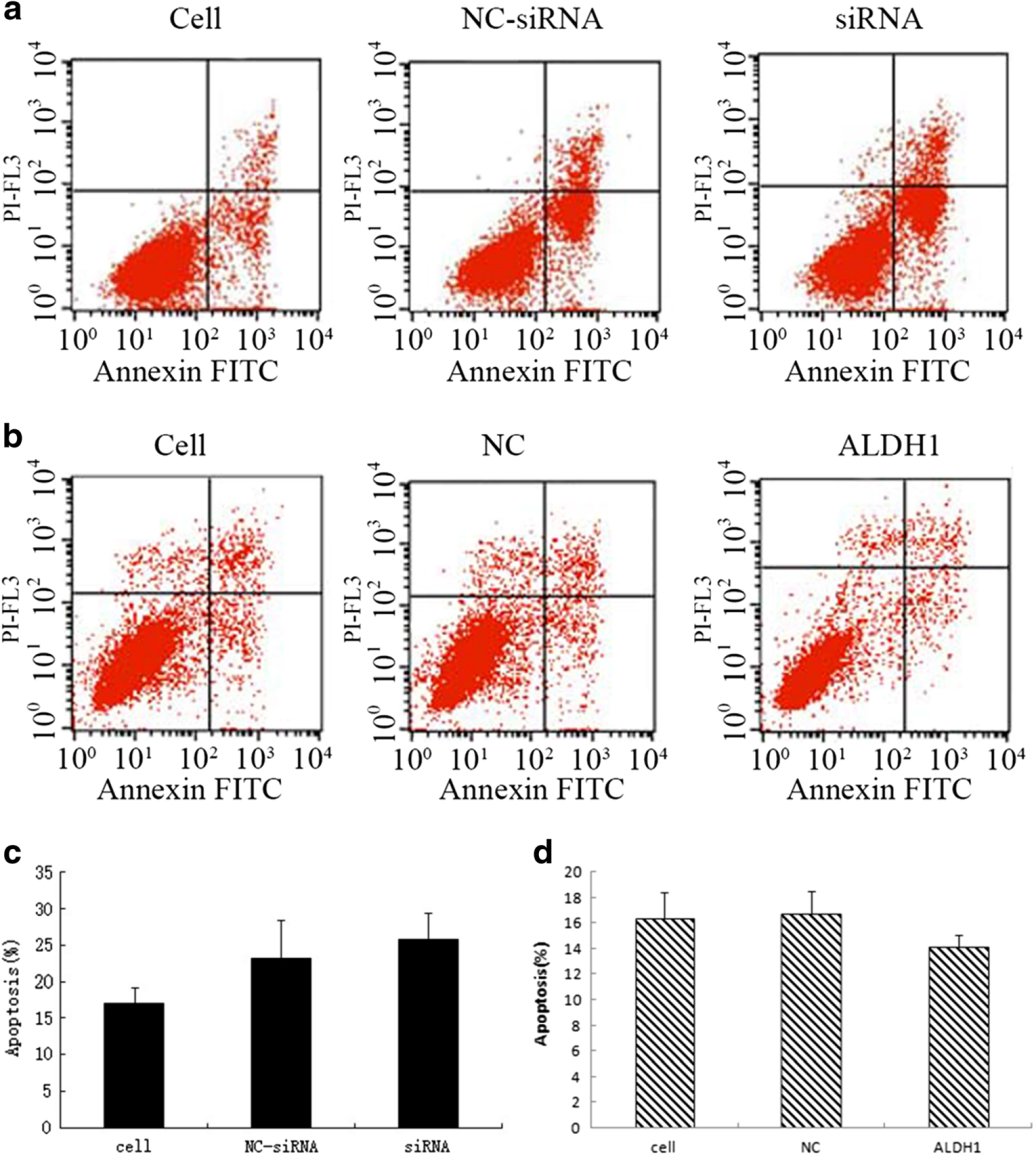
Cell death was monitored by Annexin V staining and flow cytometry. The right lower quadrant of each plot contains early apoptotic cells, whereas the right upper quadrant contains late apoptotic cells. This experiment was repeated on three independent occasions, and similar results were obtained each time. **a** The cells without any treatment, cells with non-targeting siRNA and cells with siRNA targeting the specific sequence. **b** The cells without any treatment, cells just with vector and cells with pIRES2/ALDH1 construct. **c** Each *bar* represents mean values ± SE from three independent experiments for cells without any treatment, with non-targeting siRNA and with siRNA targeting the specific sequence. **d** Each *bar* represents mean values ± SE from three independent experiments for cells without any treatment, cells just with vector and cells with pIRES2/ALDH1 construct

**Fig. 4:**
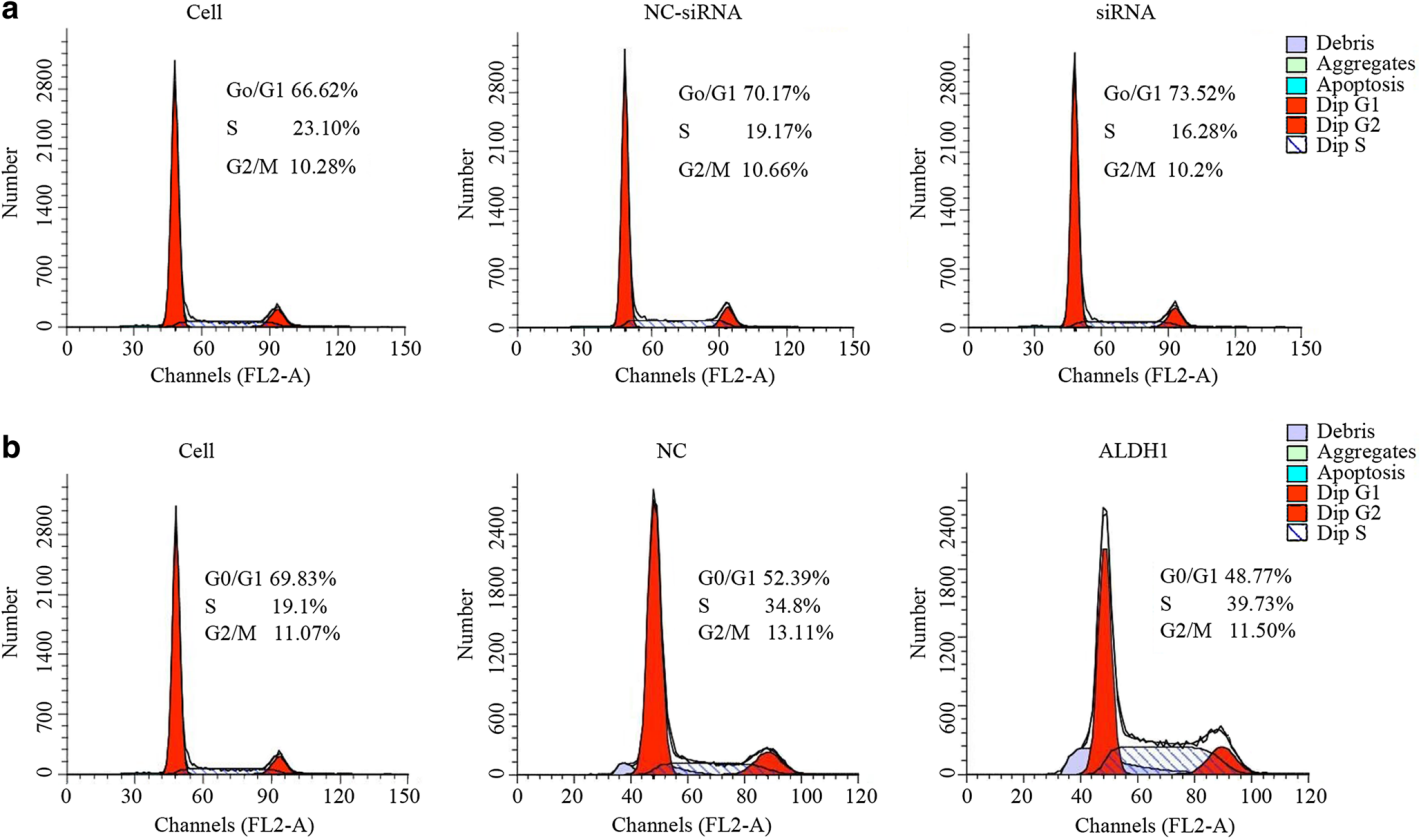
The cell cycle distribution of the cells was analyzed by FACS. **a** The cells without any treatment, cells with non-targeting siRNA and cells with siRNA targeting the specific sequence. **b** The cells without any treatment, cells just with vector and cells with pIRES2/ALDH1 construct

As shown in Fig. 2a, knockdown of ALDH1 using the oligonuclear sequences resulted in no significant decrease and no significant increase in the HeLa cell proliferation (Fig. 2a), apoptosis (Fig. 3a, c), and cell cycle (Fig. 3a) compared to the empty vector-transfected cells. Similarly, no obvious alteration of the HeLa cells was found in the ALDH1 overexpressing cells in comparison to that in the control sequence-transfected group (Figs. 2b, 3b, c, and 4b).

### ALDH1 influences HeLa cell migration in vitro

**Fig. 5:**
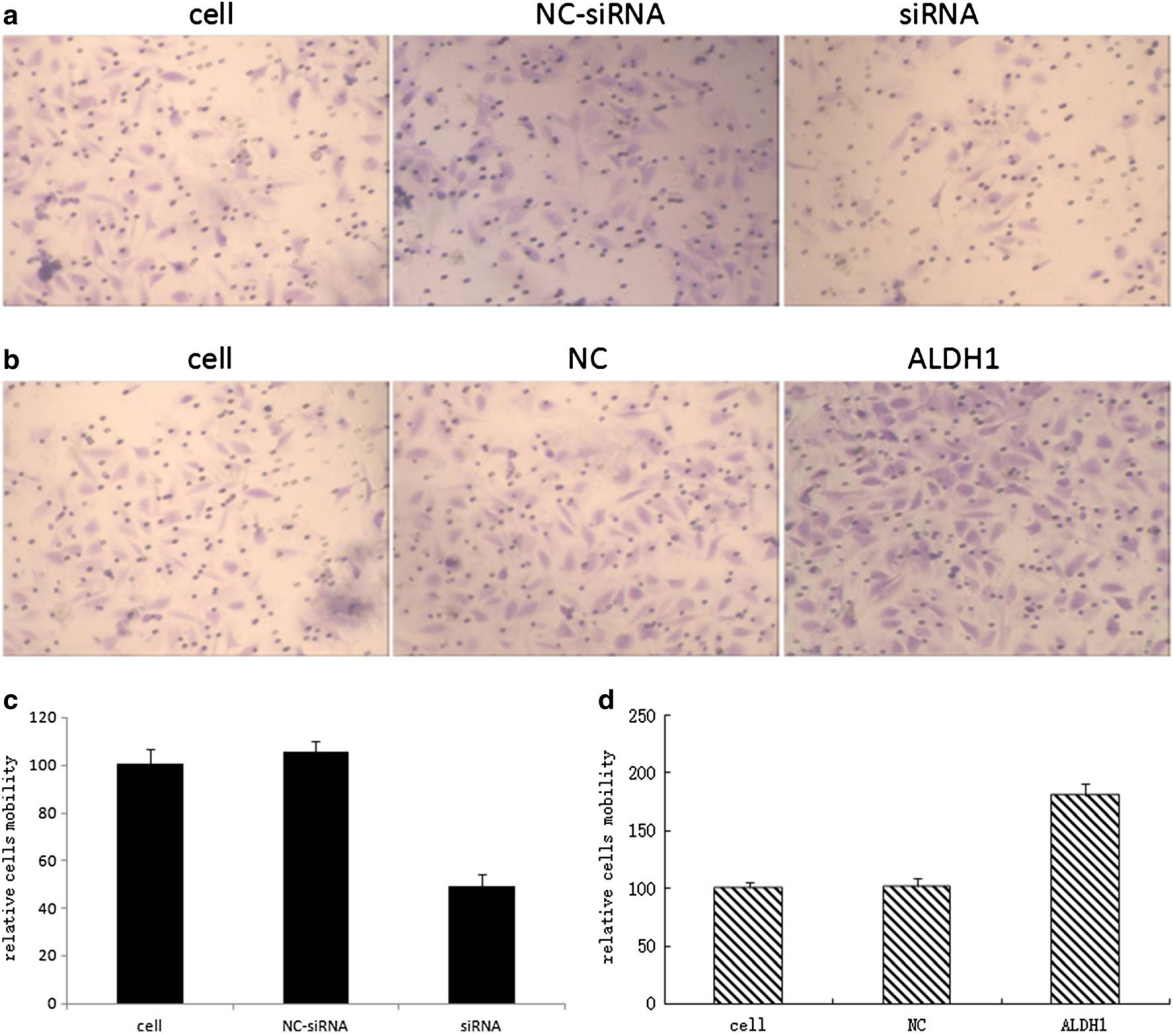
The Transwell analysis of migration. **a** The cells without any treatment, cells with non-targeting siRNA and cells with siRNA targeting the specific sequence. **b** The cells without any treatment, cells just with vector and cells with pIRES2/ALDH1 construct. **c** Each *bar* represents mean values ± SE from three independent experiments for cells without any treatment, with non-targeting siRNA and with siRNA targeting the specific sequence. **d** Each *bar* represents mean values ± SE from three independent experiments for cells without any treatment, cells just with vector and cells with pIRES2/ALDH1 construct

To examine the effect of ALDH1 on the mobility of HeLa cells, the migration was measured by Transwell assays. We showed that the SiRNA-transfected HeLa cells had lower migration rates than the control (Fig. 5a, c), whereas the HeLa-ALDH1-positive cells had significantly higher migration rates than their control cells (Fig. 5b, d), suggesting that ALDH1 is an effective target for treating cervical cancer metastases.

## Discussion

Recurrence and metastasis are the most important factors affecting the prognosis of patients with cervical cancer [18]. Developing molecular biomarkers to detect cancer, predicting disease progression, and monitoring therapeutic response would be beneficial. ALDH1 is a detoxifying enzyme responsible for the oxidation of retinol to retinoic acid, and it has a role in the early differentiation of stem cells.

Recent studies showed that ALDH1 expression was associated with tumor cell metastasis in vivo [19, 20]. According to the report of the Sidney Kimmel Comprehensive Cancer Center in the USA, a high ALDH expression is associated with worse overall survival in patients who have undergone resection for early-stage disease. The European Society for Medical Oncology (ESMO) has established Practice Guidelines that suggest that enhanced clonogenic growth of ALDH+ cancer cells has an important role in the long-term outcome of patients diagnosed with pancreatic adenocarcinoma by mediating or even stimulating cancer dissemination throughout the abdominal cavity and resistance to chemotherapy with 5-fluorouracil (5-FU) or gemcitabine [21].

To confirm whether ALDH1 was involved in cervical cancer metastasis, we performed a Transwell migration assay in vitro to investigate the effect of ALDH1 on HeLa cell mobility. Similar to those of previous studies, our results showed that the migrated rate of cells was concordant with ALDH1 expression. However, our results indicated that ALDH1 has no relationship with cell proliferation, which was not consistent with previous reports. Additionally, we found that the rates of apoptotic cells and cell cycle distribution did not present any significant alteration. The finding suggested that ALDH1 might participate in the tumorigenesis of cervical cancer through pathways other than the promotion of cell growth and obstruction of apoptosis. ALDH1 might be involved in metastasis in cervical cancer patients. ALDH1 could be used as a therapeutic target.

## Conclusion

Our results demonstrated that ALDH1 might be involved in cervical cancer metastasis. Further experiments in vivo are necessary to clarify that ALDH1 might be a promising target and prognostic predictor for treating cervical cancer metastases.
